# Single-subject auditory ERP-BCI performance enhancement in ALS via an AI coding assistant prompt

**DOI:** 10.3389/fnhum.2026.1869918

**Published:** 2026-07-01

**Authors:** Mikito Ogino

**Affiliations:** Graduate School of Arts and Sciences, The University of Tokyo, Tokyo, Japan

**Keywords:** brain–computer interface, auditory event-related potential, amyotrophic lateral sclerosis, single-subject optimization, large language model, linear discriminant analysis, information transfer rate

## Abstract

**Introduction:**

Auditory event-related potential (ERP) brain-computer interfaces (BCIs) offer communication support for individuals with amyotrophic lateral sclerosis (ALS) who eventually progress to completely locked-in states. However, individual-specific BCI pipeline optimization is technically demanding and time-consuming, leaving substantial room for performance improvement in practice. A central challenge is increasing selection speed while maintaining reliable classification accuracy, since slower selections reduce the sense of agency and undermine the motivational and feedback dynamics essential for sustained BCI use.

**Methods:**

We investigated whether an AI coding assistant could address this challenge for individual patients. A three-class auditory ERP-BCI was optimized for a single ALS patient using Claude Code (Anthropic, Inc.), which iteratively generated and evaluated 23 optimization scripts over approximately 24 hours with minimal human-in-the-loop oversight. The resulting AI-Designed ERP classifier (AIDE) was evaluated on 189 EEG trials spanning 3.5 years using five cross-validation strategies.

**Results:**

For the baseline models, halving the stimulus repetitions to shorten selection time degraded classification accuracy; AIDE prevented this degradation, achieving 85.03% mean cross-validation accuracy (selection time 17 s; ITR 2.92 bits/min). This doubled the information transfer rate from 1.43 to 2.92 bits/min. Accuracy exceeded 84% across four of five cross-validation strategies. Feature space visualization revealed that the AI autonomously selected and combined EEG features established in prior studies into an effective discriminative architecture, without domain-specific algorithmic guidance from the human researcher. In addition, online test confirmed 66.7% accuracy for AIDE versus 50.0% for the baseline model.

**Discussion:**

These findings provide proof of concept that single-subject BCI performance can be improved via a single prompt, offering an efficient pathway to individualized optimization in clinical and research settings.

## 1 Introduction

**Figure 1:**
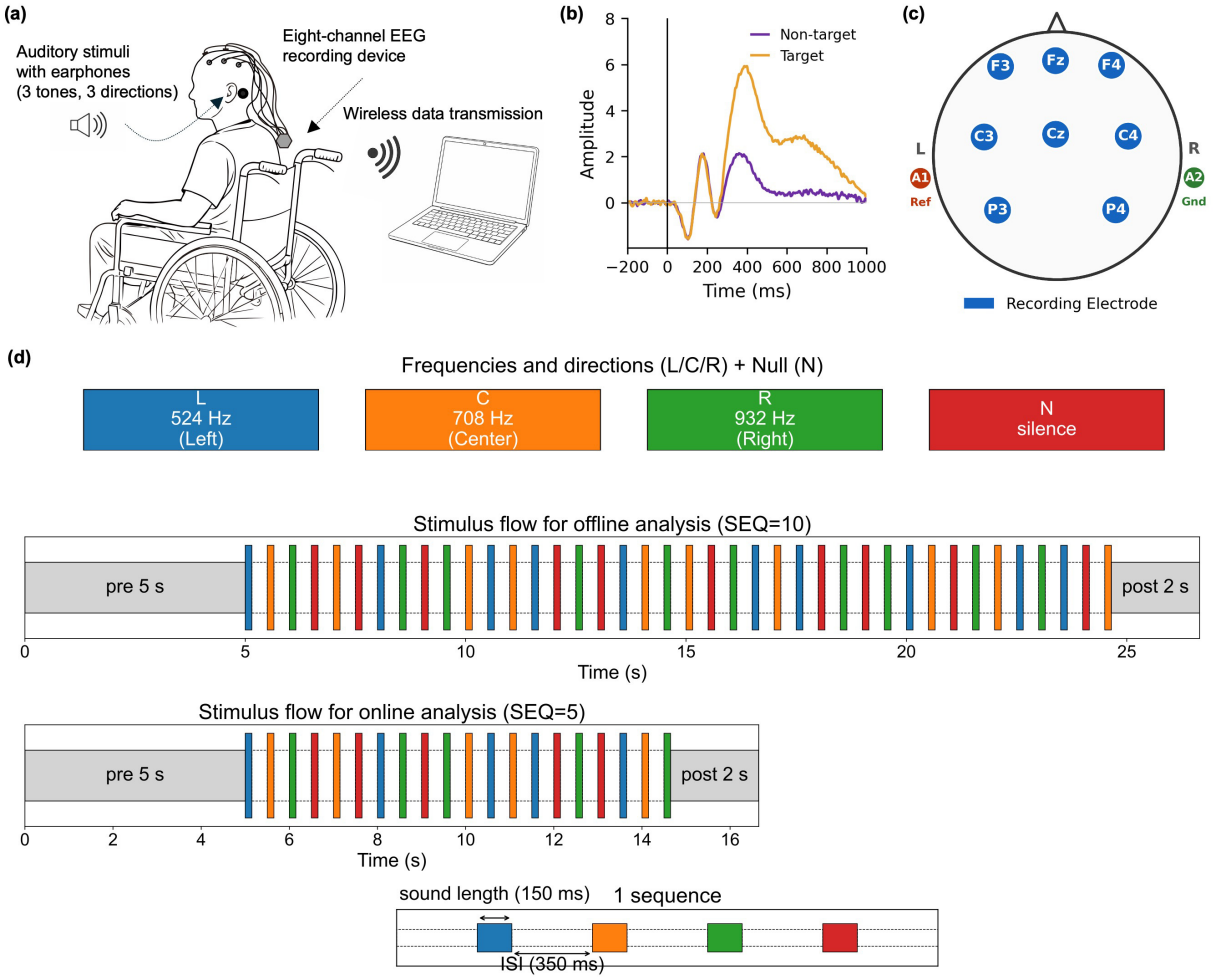
Study overview: participant setup, representative ERP, electrode placement, and auditory stimulus paradigm. **(a)** An ALS patient seated in a wheelchair with an 8-channel EEG headset (OpenBCI Cyton) mounted, illustrating the clinical recording context in which all sessions were conducted. **(b)** Representative grand-average event-related potentials (ERPs) at electrode Fz for target (yellow line) and non-target (purple line) stimuli, showing the characteristic late positive deflection that distinguishes attended from unattended auditory stimuli. **(c)** Top-view schematic of the 8-channel EEG electrode placement following the international 10–20 system. **(d)** Schematic of the auditory stimulus flow. The upper timeline shows the offline recording protocol (SEQ = 10): each trial consists of a 5 s pre-stimulus silence, 10 randomized repetitions of the four sound classes (Left L, Center C, Right R, and Null N), and a 2 s post-stimulus silence. The lower timeline shows the online test protocol (SEQ = 5): the same four-class randomized sequence is presented with five repetitions per trial, yielding a decision every 17 s. The zoom inset illustrates the timing of individual stimuli in a single sequence (sound length 150 ms, inter-stimulus interval 350 ms).

Brain–computer interfaces enable direct communication between the human nervous system and external devices (Wolpaw et al., 2002; Vansteensel et al., 2016). They provide a communication pathway for individuals with severe motor impairments such as amyotrophic lateral sclerosis (ALS). ALS progressively destroys motor neurons and ultimately deprives patients of all voluntary movement including eye-gaze control in advanced stages. At this stage, conventional gaze-based BCIs become unusable. Auditory event-related potential (ERP) BCIs address this limitation by operating entirely through the auditory modality, which requires no motor output or visual fixation (Klobassa et al., 2009; Halder et al., 2010; Séguin et al., 2024). In such systems, the user covertly attends to one target tone in a multi-stimulus auditory sequence; the resulting ERP, an auditory analog of the visual P300 component, is decoded to infer the attended target (Farwell and Donchin, 1988; Furdea et al., 2009; Höhne et al., 2010) (Figure 1b). Despite their clinical importance, auditory ERP-BCIs have received less attention than visual systems, and their classification accuracy and information transfer rate (ITR) typically remain below practical thresholds for daily use.

A persistent challenge in auditory ERP-BCI research is to reduce selection time (defined as the number of stimulus repetitions required per decision) without sacrificing classification accuracy. Rapid feedback is essential for sustaining user engagement and supporting communication practice (Kleih et al., 2010; Kleih and Kubler, 2015); however, shorter selection times inherently degrade signal quality, as ERP detection relies on averaging across repetitions to suppress noise. Therefore, improving selection speed requires careful optimization of the signal processing pipeline, which includes multiple interdependent parameters such as filter cutoffs, epoch durations, channel subsets, downsampling factors, feature extraction modes, and classifier hyperparameters. They interact in complex ways, and exhaustive grid search over the full parameter space is computationally prohibitive. Crucially, optimal parameters vary substantially across individuals due to anatomical, physiological, and habituation differences, meaning that a pipeline tuned for one subject may perform poorly for another (Saha and Baumert, 2019; Fathima and Kore, 2020).

To address this challenge, we explored whether an AI coding assistant could optimize an auditory ERP-BCI pipeline for an individual user without domain-specific guidance from the human researcher. Recent studies have shown that large language models (LLMs) can write, debug, and iteratively improve scientific code across diverse domains (Poldrack et al., 2023; Chen et al., 2023; Zhuang and Lin, 2024). In this paper, we report a single-subject case study in which Claude Code (Anthropic, Inc.) autonomously optimized the signal processing pipeline of a three-class auditory ERP-BCI for one ALS patient, using that individual's longitudinal EEG (electroencephalography) dataset. We optimized the pipeline to maximize mean stratified cross-validation accuracy at approximately half the original selection time without any further human input after the initial prompt. The resulting AI-Designed ERP classifier (AIDE) was evaluated on 189 EEG trials collected across 10 recording sessions spanning approximately 3.5 years, using five cross-validation strategies to characterize model robustness and longitudinal stability. The AI autonomously selected and combined standard EEG feature constructs into an effective architecture that halved the required stimulus repetitions for reliable performance.

The remainder of this paper is organized as follows. Section 2 describes the participant, EEG acquisition protocol, auditory stimulus paradigm, the dataset, the baseline signal processing pipeline, the LLM-assisted optimization procedure, and the offline and online evaluation protocols. Section 3 presents the auditory ERP waveforms, the LLM development timeline, offline cross-validation accuracy across five strategies, feature space visualizations, the effect of the number of stimulus repetitions on classification accuracy, and online test results. Section 4 interprets these findings in the context of auditory ERP-BCI research and discusses the implications of LLM-driven pipeline optimization. Section 5 summarizes the main contributions and outlines directions for future work.

## 2 Materials and methods

### 2.1 Participant

One individual with amyotrophic lateral sclerosis (ALS; male, right-handed; age 35 years at initial recording in 2022, 39 years at final recording in 2026) participated in EEG data collection across 10 sessions spanning 2022 to 2026. The participant retained fine voluntary movements in both hands, enabling sensor-based operation of a wheelchair and communication device click actions. He was able to use a keyboard for communication via an eye-tracking input device, and could also communicate by moving his mouth or using a letter board. The participant's Amyotrophic Lateral Sclerosis Functional Rating Scale (ALSFRS) score was 12 at the initial recording session in 2022 and 11 in all subsequent sessions. The 2022 recording sessions were approved by the Ethical Review Committee of Dentsu ScienceJam Inc. (approval number 006). The 2025–2026 recording sessions were approved by the Ethics Review Committee for Human Experiments at the Graduate School of Arts and Sciences, The University of Tokyo (approval number: 996). Written informed consent was obtained from the participant prior to each recording session. All procedures were conducted in accordance with the Declaration of Helsinki.

### 2.2 EEG acquisition

EEG acquisition was performed using the Cyton Biosensing Board (OpenBCI) (Cano et al., 2020; Shivaraja et al., 2025) (Figure 1a). The system recorded neural signals at a sampling frequency of 250 Hz, with eight channels each digitized at 24-bit resolution, and transmitted the acquired data wirelessly to a host computer. Electrode attachment followed a passive configuration, with conductive paste applied at each site to ensure stable scalp-electrode contact. Recording sites were selected from the international 10–20 system, comprising F3, Fz, F4, C3, Cz, C4, P3, and P4. The left earlobe (A1) served as the reference, and the right earlobe (A2) as the ground. The spatial distribution of all electrodes across the frontal, central, and parietal regions is depicted in Figure 1c.

### 2.3 Auditory stimulus paradigm

The acoustic stimulation protocol is summarized in Figure 1d. Three pure tones at 524 Hz, 708 Hz, and 932 Hz were spatially assigned to the left (L), center (C), and right (R) directions, respectively. Each tone was 150 ms in duration, separated by 350 ms inter-stimulus intervals. Following the oddball paradigm, a 150 ms silent epoch (N) was embedded in the stimulus sequence to augment ERP amplitude (Duncan-Johnson and Donchin, 1977; Polich, 2007). To issue a command, participants focused their attention on one of the three target tones. Attentional engagement was further reinforced by coupling each stimulus direction with motor imagery: left-hand movement for the left tone, foot movement for the center tone, and right-hand movement for the right tone (Long et al., 2017; Zuo et al., 2020). A single trial consisted of 10 sequences, preceded by a 5 s pre-stimulus silence and followed by a 2 s post-stimulus silence, yielding a total trial duration of 27 s, within which the participant was required to complete one selection.

### 2.4 Experimental procedure

A total of 189 trials were collected across 10 recording sessions spanning 2022 to 2026 (approximately 3.5 years). The first five sessions were conducted in 2022, yielding 135 trials in total (18, 27, 36, 36, and 18 trials per session, respectively). After a gap of approximately 2.5 years reflecting the progression of the participant's condition and a shift toward clinical BCI deployment, five additional sessions were conducted in 2025–2026, yielding 54 further trials (15, 6, 6, 12, and 15 trials, respectively). The later sessions contain fewer trials per session, reflecting the increasing effort required for the participant as motor function declined.

### 2.5 Baseline system

**Figure 2:**
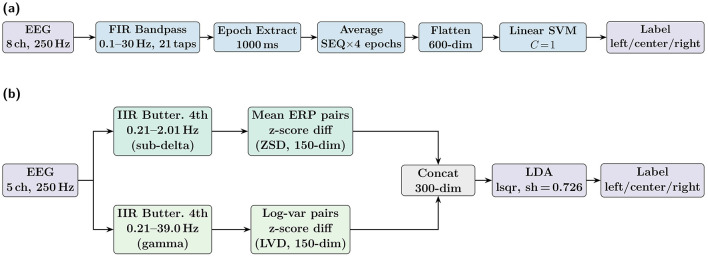
Signal processing pipelines compared in this study. **(a)** Baseline pipeline using a single FIR bandpass filter and linear SVM, requiring SEQ = 10 for reliable operation. **(b)** AI-optimized AIDE pipeline: purple = input/output; blue = baseline processing steps; teal = sub-delta band; green = gamma band.

The signal processing pipeline of the baseline system is illustrated in Figure 2a. The baseline system was built upon prior works (Simon et al., 2014; Ogino et al., 2019, 2022). The pipeline consists of a bandpass FIR filter (0.1–30 Hz, 21 taps), epoch extraction (1,000 ms post-stimulus), averaging across epochs, flattening to a 600-dimensional feature vector, and classification using a linear support vector machine (SVM) (*C* = 1). The baseline LASSO system uses the same feature extraction but replaces the classifier with a one-vs-rest LASSO regressor (α = 0.1, standardized features). A third baseline (Baseline LDA) applies linear discriminant analysis [LDA; Ledoit–Wolf auto-shrinkage (Ledoit and Wolf, 2004)] to the same feature extraction. These baselines required 10 stimulus sequences (SEQ) per trial for reliable performance.

### 2.6 LLM-assisted optimization

#### 2.6.1 Accuracy evaluation protocol

We used the mean stratified cross-validation accuracy to prevent overfitting to specific random seeds or data splits during optimization. This protocol computes the mean accuracy across 10 random seeds of stratified *k*-fold cross-validation (*k* ∈ {3, 4}) with undersampling to balance class proportions; accuracy was averaged across all folds and seeds to estimate in-distribution generalization. The LLM was instructed to keep this accuracy computation method fixed during optimization.

#### 2.6.2 Initial prompt and objective

The researcher entered a single prompt in Japanese, providing Claude Code with the optimization objective, the data location, the constraints (SEQ fixed at 5, evaluation protocol unchanged), and the stopping criterion (mean accuracy > 0.85). The Japanese original is reproduced in Supplementary material S1. An English translation is provided below:

**English translation (see** **Supplementary material S1** **for the Japanese original):** “*Copy [the existing BCI classification script] and run auditory ERP BCI analysis to maximize the Mean accuracy (mean stratified cross-validation accuracy) that the script prints. The target data is all files in the data folder. First investigate how the existing script conducts the analysis; SEQ = 5 is fixed. SEQ = 10 gives higher accuracy but is slow, so the goal is to increase speed. Keep the Mean accuracy computation method fixed and continue analysis until mean accuracy exceeds 0.85. Install pip packages automatically. Allow all executions so the process does not stop. Output the accuracy improvement history with timestamps and methods used to a CSV file.”*

No further algorithmic instructions were provided by the researcher after this prompt.

#### 2.6.3 Human involvement

The total human text input during the 24-h development period was minimal and non-substantive. Throughout the session, the researcher responded “YES” to each tool-use permission prompt issued by Claude Code (e.g., permission to read data files, execute Python scripts, or write new files); no substantive decision-making was involved in these approvals. On one occasion, when the daily API token limit was reached, the researcher entered a brief instruction to pause and restart: “*Please wait a moment and then restart the process*.” The AI then resumed the optimization from where it had stopped. No manual code writing, parameter selection, or algorithmic reasoning was performed by the human researcher after the initial prompt.

### 2.7 The AI-Designed ERP classifier (AIDE)

Figure 2 compares the baseline pipeline (a) and the pipeline of the AI-designed ERP classifier (AIDE) (b). This section describes the optimized pipeline. The AI employed Optuna for hyperparameter optimization; the complete search space is detailed in the Supplementary material S3.

#### 2.7.1 Preprocessing

EEG data from five selected channels (F3, Fz, F4, C3, C4) were extracted and multiplied by −1 to correct hardware polarity inversion. The channel-subset selection is justified in Supplementary material S4. Channels were z-score standardized (zero mean, unit variance) across the full recording. No common average reference (CAR) was applied (determined suboptimal by the Optuna search).

Two fourth-order Butterworth bandpass filters were applied using zero-phase (forward–backward) filtering: a low band of 0.21–2.01 Hz capturing sub-delta slow cortical potentials, and a high band of 0.21–39.0 Hz extending up to the gamma range.

#### 2.7.2 Epoch extraction and averaging

For each stimulus onset (up to 3 × SEQ per trial), a 1000-ms epoch (250 samples) was extracted starting at onset. No pre-stimulus baseline correction was applied (bl = 0, confirmed optimal by Optuna). Epochs were grouped by stimulus label (Left, Center, or Right), and the per-label mean ERP ${\bar{\text{x}}}_{l}$ and standard deviation ***σ***_*l*_ were computed. Trials in which the maximum absolute amplitude of the averaged ERP exceeded 100 μV in any channel were excluded as artifacts prior to feature extraction.

#### 2.7.3 Feature extraction

Features were extracted for all (32) = 3 label pairs using two complementary modes. For the sub-delta band, a *z*-scored mean difference (ZSD) was computed (Equation 1):


$$\begin{array}{l} {\text{f}}_{ij}^{\text{low}}=\text{zscore}\left(\text{down}\left({\bar{\text{x}}}_{i}-{\bar{\text{x}}}_{j}\right)\right), \end{array}$$ (1)

where down(·) denotes mean-pooling into *ds* = 10 non-overlapping temporal windows of *W*/*ds* = 250/10 = 25 samples each, where *W* = 250 is the epoch length in samples; this captures ERP waveform shape differences related to sustained auditory attention. For the gamma band, a log-scale variance difference (LVD) was computed (Equation 2):


$$\begin{array}{l} {\text{f}}_{ij}^{\text{high}}=\text{zscore}\left(\text{down}\left(\log\sigma_{i}-\log\sigma_{j}\right)\right), \end{array}$$ (2)

where the log transformation linearizes multiplicative gamma-band power dynamics, which follow a log-normal distribution. This makes the feature better suited to the Gaussian assumption of LDA. The full feature vector for one trial is then (Equation 3):


$$\begin{array}{l} \text{x}={\left[{\text{f}}_{12}^{\text{low}},{\text{f}}_{13}^{\text{low}},{\text{f}}_{23}^{\text{low}},{\text{f}}_{12}^{\text{high}},{\text{f}}_{13}^{\text{high}},{\text{f}}_{23}^{\text{high}}\right]}^{T}\in {\mathbb{R}}^{300}, \end{array}$$ (3)

with 3 pairs × 10 windows × 5 channels × 2 bands = 300 dimensions.

#### 2.7.4 Classification

LDA with Ledoit–Wolf shrinkage (Ledoit and Wolf, 2004) was used: solver = lsqr, shrinkage = 0.726 (optimized by Optuna). Shrinkage regularizes the covariance estimate when the number of samples (*n* = 189) is smaller than the number of features (*d* = 300) (Blankertz et al., 2011).

#### 2.7.5 Evaluation

Five cross-validation strategies were employed to assess generalizability from multiple perspectives. The first, StratifiedKFold (*k* ∈ {3, 4}, 10 random seeds), performed standard stratified *k*-fold cross-validation repeated across 10 random seeds, with the training set undersampled at each fold to balance class proportions; accuracy was averaged across all folds and seeds to estimate in-distribution generalization. The second, day-out leave-one-session-out (LODO), held out each of the 10 recording sessions in turn as the test set, using all remaining sessions for training, thereby assessing generalization across recording days and susceptibility to session-to-session EEG non-stationarity. The third, leave-one-trial-out (LOTO), applied strict leave-one-out over all 189 individual trials; because each test set contains exactly one trial, fold-level scores are binary, and mean accuracy equals the proportion of correctly classified trials. The fourth, temporal split, trained the classifier exclusively on the 135 trials from 2022 and tested on the 54 trials from 2025–2026, evaluating whether the model degrades over a multi-year gap without recalibration and reflecting the clinically realistic scenario of long-term deployment. The fifth, day GroupKFold (*k* = 5), used five-fold cross-validation in which folds were constructed to keep all trials from the same session in the same partition, preventing data leakage across days; fold assignment is deterministic given the session structure.

The accuracy change as a function of the number of stimulus repetitions (SEQ) was also evaluated. The baseline and proposed models were evaluated with SEQ = 1 through 10. For each value, mean accuracy was computed across stratified *k*-fold cross-validation (*k* ∈ {3, 4}, 10 random seeds) with undersampling to balance class proportions.

### 2.8 Online test protocol

To evaluate actual BCI performance under realistic conditions, an online test was conducted in 2026 after all offline analyses were completed. Each model was trained using all data collected during the offline evaluation. The session consisted of four blocks of nine trials each, alternating between the baseline model and the AIDE (baseline, AIDE, baseline, AIDE). EEG signals were acquired and classified in real time, and the decoded selection was immediately displayed on the screen and verbally communicated to the participant. The participant was blinded to which model was active at each block.

### 2.9 Information transfer rate

ITR was computed as Wolpaw et al. (2002) (Equations 4, 5):


$$\begin{array}{l} B=\log_{2}N+P\log_{2}P+\left(1-P\right)\log_{2}\frac{1-P}{N-1}\;\text{[bits/selection]}, \end{array}$$ (4)

$$\begin{array}{l} \text{ITR}=\frac{B\times 60}{T}\;\text{[bits/min]}, \end{array}$$ (5)

where *N* = 3 (classes), *P* is accuracy, and *T* = 5+4 × 0.5 × SEQ+2 = (7+2SEQ) s is the full trial duration, comprising the 5 s pre-stimulus silence, the stimulus sequence, and the 2 s post-stimulus silence.

## 3 Results

### 3.1 Auditory ERP waveforms

**Figure 3:**
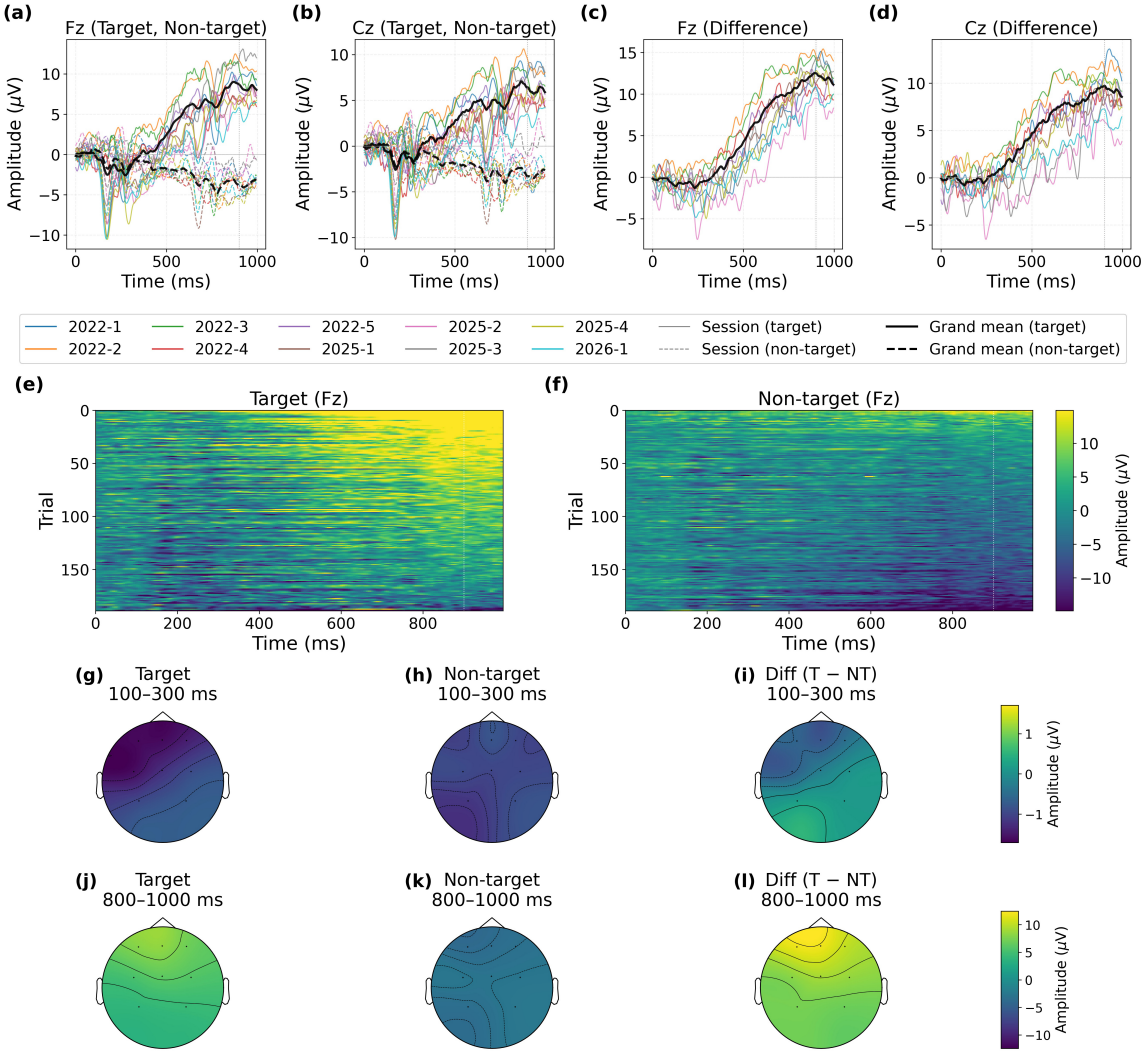
Auditory ERP consistency across 189 trials. Thin lines show individual trial-level means; thick lines show the grand mean across trials. **(a)** Fz: target (solid) vs. non-target (dashed). **(b)** Cz: target vs. non-target. **(c)** Fz difference (Target − Non-target). **(d)** Cz difference. Vertical dotted lines in **(a–d)** mark the peak-difference latency. Session colors are labeled YYYY-*N* in the legend, where *N* is the chronological index of that session within the calendar year (e.g., 2022-1 is the first session recorded in 2022). **(e)** ERP image at Fz for target trials; each row is one sequence-averaged trial sorted by mean amplitude in a ±100 ms window around the peak latency (strongest response at top). Color encodes raw EEG amplitude. **(f)** Same for non-target trials. **(g–i)** Scalp topographies averaged over 100–300 ms post-stimulus onset (N2 window): target mean, non-target mean, and difference. **(j–l)** Scalp topographies averaged over 800–1000 ms (late positive complex window): target mean, non-target mean, and difference.

Figure 3 summarizes the event-related potential (ERP) recorded across all 189 trials (1890 target, 3780 non-target epochs after pooling across the three attended stimulus classes). Grand-average waveforms at Fz and Cz (Figures 3a, b) show a clear negativity around 100–300 ms (N2) followed by a sustained positive deflection peaking at the latency of maximal target/non-target divergence. Figures 3c, d show the Fz and Cz difference waveforms (Target − Non-target), respectively, with a vertical dotted line marking the peak-difference latency. Figures 3e, f display ERP images at Fz for target and non-target trials, respectively: each row is one sequence-averaged trial and the color encodes the raw EEG amplitude. Trials are sorted in descending order of mean Fz amplitude within a ±100 ms window centered on the peak-difference latency (900 ms), so that trials with the strongest ERP response appear at the top. This ordering reveals a consistent late positive deflection across the majority of target trials that is largely absent in non-target trials. Scalp topographies averaged over 100–300 ms (Figures 3g–i) capture the N2 auditory response, while topographies over 800–1000 ms (Figures 3j–l) reflect the sustained late positive complex; both confirm a frontocentrally dominant target response with maximum amplitude at Fz–Cz.

### 3.2 Development process

Starting from the existing baseline code, Claude Code generated 23 successive optimization scripts (Supplementary material S2), each building on quantitative insights from the preceding run. The AI independently introduced Bayesian hyperparameter optimization using Optuna (Akiba et al., 2019) with Tree-structured Parzen Estimator (TPE) sampling, and explored a multi-dimensional search space spanning bandpass filter cutoff frequencies, feature extraction modes, EEG channel subsets, temporal downsampling factors, and LDA regularization parameters. The complete search space is detailed in the Supplementary material S3. Candidate configurations were evaluated via stratified *k*-fold cross-validation (*k* ∈ {3, 4}, 10 random states, stratified undersampling of the training set), and all results together with the corresponding parameter settings were recorded to CSV log files throughout the run. Key algorithmic milestones and the accuracy achieved at each stage are summarized in Table 1. The optimization spanned approximately 24 hours. The AI completed all stages of pipeline exploration with minimal researcher input, which consisted of granting tool-use permissions during the run and issuing one brief instruction to resume after the daily API token-limit reset. Accuracy increased from 79.75% to 85.03% over this period.

**Table 1:** Key milestones in the LLM-driven optimization process.

Script version	Key discovery	Accuracy
v2 (start)	Baseline SVM, single-band FIR (0.1–30 Hz)	79.75%
v11	Mean + variance difference feature (MVD)	81.38%
v17	Two-band approach; gamma variance difference (VD)	83.21%
v18	Channel subset F3, Fz, F4, C3, C4; SEQ = 5 features	84.00%
v22	Log-scale gamma variance (LVD) mode added	83.81%
v23 (final)	Optuna Bayesian search: sub-delta + gamma + lsqr LDA	**85.03%**

Bold indicates the accuracy of the final adopted model.

### 3.3 Offline cross-validation accuracy

The optimized model achieved 85.03% (±0.010 SD across random states). Table 2 compares the AIDE against both baselines (existing pipeline) across all five strategies at SEQ = 5.

**Table 2:** Cross-validation accuracy at SEQ = 5 for the AIDE and the three baselines.

Strategy	AIDE	Baseline SVM	Baseline LASSO	Baseline LDA
StratifiedKFold (*k* = 3,4; 10 seeds)	85.03%	70.11%	66.90%	67.71%
Day-out (LODO, 10 sessions)	84.96%	73.63%	68.00%	68.15%
One-trial-out (LOTO, 189-fold)	85.19%	72.49%	67.20%	67.20%
Temporal split (2022 → 2025–26)	77.78%	70.37%	57.41%	68.52%
Day GroupKFold (*k* = 5)	84.06%	68.25%	65.73%	68.76%

**Figure 4:**
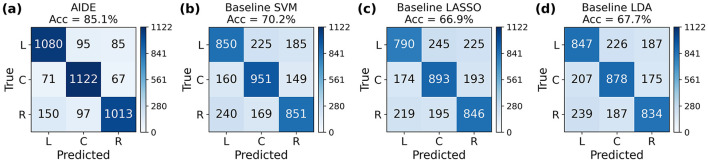
Balanced multi-class predictions in StratifiedKFold cross-validation. Raw-count confusion matrices aggregated over 10 seeds × *k* ∈ {3, 4} cross-validation folds. Each cell shows the number of trials of the true class (row) predicted as the column class. **(a)** AIDE, **(b)** baseline SVM, **(c)** baseline LASSO, **(d)** baseline LDA.

The three standard strategies (StratifiedKFold, LODO, LOTO) yielded virtually identical mean accuracies for the AIDE (85.0%–85.2%), indicating stable performance regardless of fold assignment method. All three baselines were substantially less accurate than AIDE: the SVM reached 70%–74%, the LASSO 66%–68%, and the Baseline LDA 67%–68%. The near-identical scores of all three baselines indicate that AIDE's accuracy advantage derives from its two-band feature architecture (ZSD + LVD), not from classifier choice. Day-out accuracy for the LDA ranged from 73.3% (2026-1) to 96.3% (2022-2) across individual sessions. The temporal split (train 2022, test 2025–2026) showed a larger accuracy drop for the baseline LASSO (57.41%) than for the baseline SVM (70.37%) or AIDE (77.78%), suggesting greater sensitivity of the LASSO classifier to longitudinal distributional shift. Figure 4 presents row-normalized confusion matrices from StratifiedKFold cross-validation as a representative example for all four classifiers, providing a visual confirmation that prediction errors are distributed across all three classes without systematic concentration in any single class. The robustness of the final configuration across the top candidate configurations from the terminal optimization stage is detailed in Supplementary material S5.

### 3.4 Feature space visualization

**Figure 5:**
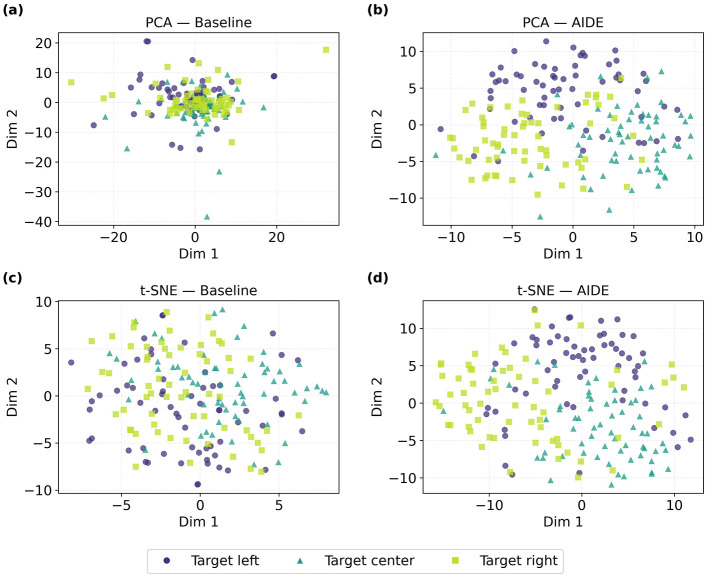
Feature space visualization of all 189 trials at SEQ = 5. Colors and markers indicate the three attended target classes. **(a)** PCA projection of the baseline feature space. **(b)** PCA projection of the AIDE feature space. **(c)** *t*-SNE projection (perplexity = 30) of the baseline feature space. **(d)** *t*-SNE projection of the AIDE feature space.

To provide geometric intuition for the accuracy gains reported in Table 2 and Figure 5 visualizes the trial-level feature spaces of the baseline SVM and the AI-optimized AIDE using two dimensionality reduction techniques: principal component analysis (PCA) and *t*-distributed stochastic neighbor embedding [*t*-SNE; (Maaten and Hinton, 2008)]. For each model, all 189 trials were projected into two dimensions from the respective feature vectors (240-dim for baseline SVM, 300-dim for AIDE).

Each scatter point in Figure 5 represents one trial and is produced by a deterministic two-stage pipeline: per-trial feature extraction from the raw EEG, followed by a 2-D embedding fitted jointly across all 189 trials within each panel. For the baseline SVM panels, the 240-dimensional feature vector of a trial is obtained by FIR band-pass filtering the raw EEG at 0.1–30 Hz, applying baseline correction, averaging the resulting ERP segments across the SEQ = 5 stimulus repetitions, downsampling the time axis by a factor of 10, and concatenating all 8 channels by 30 time points. For the AIDE panels, the same trial recording is processed by the two-band two-band pipeline: the sub-delta (0.21–2.01 Hz) and gamma-band (0.21–39 Hz) sequence-averaged ERPs are extracted at the 5 selected channels and time points, and the per-pair (left–center, left–right, center–right) *z*-scored sub-delta differences (ZSD, 150 dims) and log-scale variance differences in the gamma band (LVD, 150 dims) are concatenated into the 300-dim vector. Before projection, all 189 feature vectors of a given model are standardized to zero mean and unit variance per dimension, so that no single high-amplitude channel or band dominates the embedding. The principal component analysis (PCA) Figures 5a, b project these standardized vectors directly into their first two principal components. The *t*-SNE Figures 5c, d first reduce the standardized vectors to 50 dimensions by PCA and then embed them with *t*-SNE (perplexity = 30, 1,000 iterations); the PCA pre-reduction suppresses high-frequency feature noise without distorting the local neighborhood structure that *t*-SNE preserves. Each point is then plotted with a marker and color determined by the attended target class parsed from the trial filename, namely a circle for Target left, a triangle for Target center, and a square for Target right, so that the geometry attributable to the attended class can be read off independently of any density or overplotting effect.

Visual inspection of the feature embeddings via PCA and t-SNE demonstrates that AIDE yields superior class discriminability compared to the baseline model. Under both projection methods, AIDE features form three distinct, well-separated clusters corresponding to the target classes, whereas the baseline projections exhibit substantial inter-class overlap. Specifically, in the PCA space, AIDE effectively captures the dominant contrast directions, while the t-SNE visualization further confirms the formation of compact, local groupings with minimal interference. These visual distributions are highly consistent with the quantitative results, where the clear spatial separation of AIDE features supports its superior classification accuracy of 85%, in contrast to the lower 70%–74% accuracy observed for the baseline model.

### 3.5 Effect of number of stimulus repetitions

**Figure 6:**
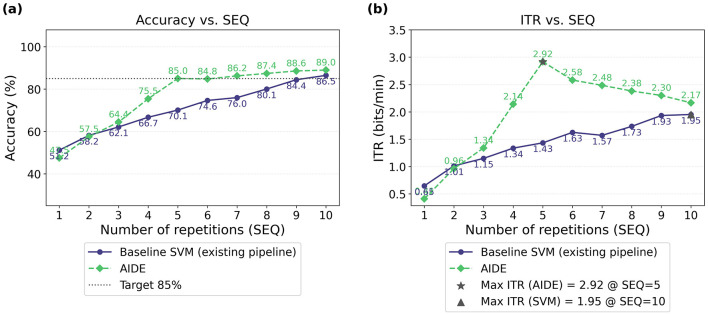
Effect of stimulus sequences on BCI performance for the AIDE and the baseline SVM. Data points are mean StratifiedKFold accuracies. **(a)** Classification accuracy vs. SEQ. The dotted line marks the 85% accuracy target. **(b)** ITR vs. SEQ. Filled markers indicate the peak ITR for each model.

Figure 6 shows classification accuracy and ITR as a function of the number of stimulus sequences for both models. The LDA model accuracy increased monotonically from 47.49% (SEQ = 1) to 88.99% (SEQ = 10), crossing the 85% threshold at SEQ = 5. The SVM baseline required SEQ = 9 to exceed 84%.

The previous system required SEQ = 10 for reliable performance. The proposed LDA model achieves 85.03% at SEQ = 5, halving the selection time. At SEQ = 5 (*P* = 85.03%), AIDE achieves a peak ITR of 2.92 bits/min; at SEQ = 10 the accuracy rises to 88.99%, but the longer decision time reduces the ITR to 2.17 bits/min. The baseline SVM at SEQ = 5 reaches only 70.11% accuracy, yielding 1.43 bits/min. Despite lower per-selection accuracy, the LDA model at SEQ = 5 yields a 35% higher ITR than the same model at SEQ = 10, and more than twice the ITR of the baseline SVM at the same repetition count.

### 3.6 Online test

**Figure 7:**
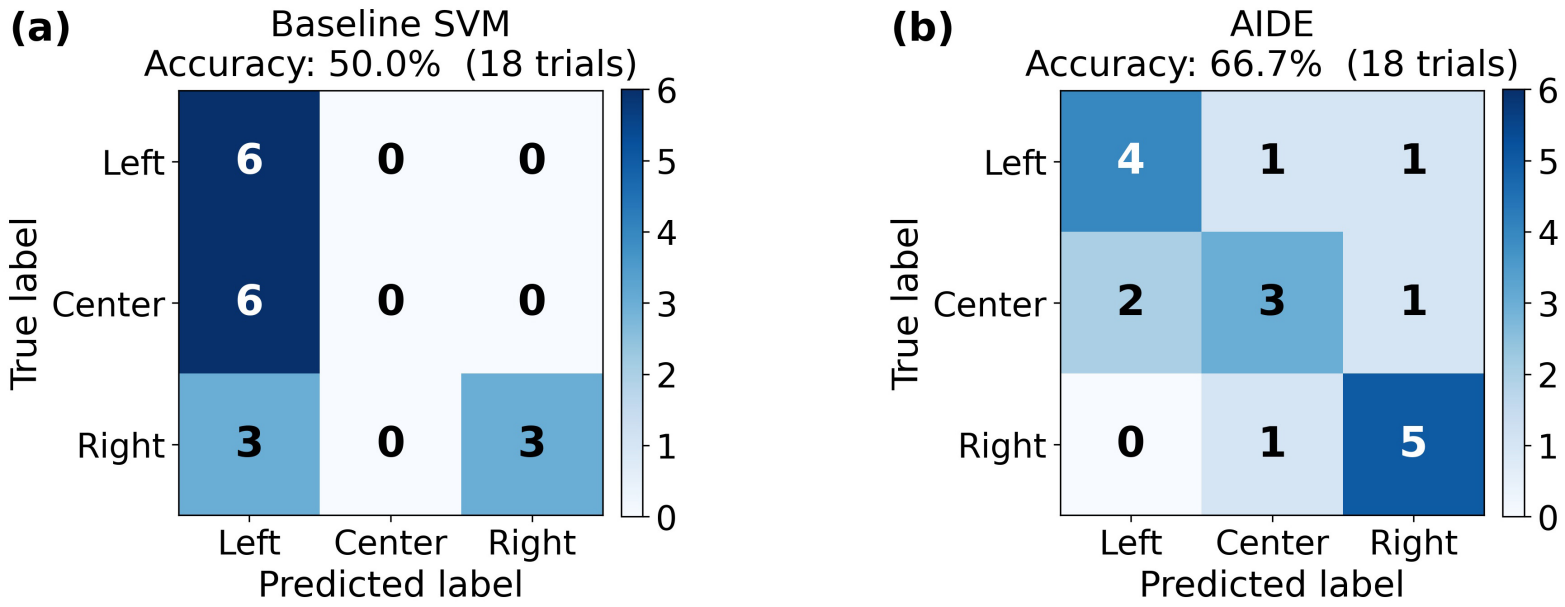
Online test confusion matrices for **(a)** baseline model and **(b)** AIDE. Each matrix shows the number of trials for each true/predicted class combination. The AIDE model achieves higher diagonal values, indicating improved real-time accuracy.

To assess real-world performance, we conducted an online test. The confusion matrices for both the baseline model and AIDE are shown in Figure 7. Each matrix summarizes the classification results across all online trials, with rows indicating the true class and columns the predicted class.

AIDE showed higher overall accuracy and fewer off-diagonal errors than the baseline. The baseline SVM predicted Left in 15 of 18 trials (83.3%), yielding perfect Left recall (6/6, 100%) but complete failure for Center (0/6, 0%) and only partial recognition of Right (3/6, 50%). AIDE produced a more balanced distribution: Left 4/6 (66.7%), Center 3/6 (50.0%), and Right 5/6 (83.3%).

The most notable gain was in Center-class recognition, which rose from 0% with the baseline to 50.0% with AIDE. Right-class recall also improved from 50.0% to 83.3%. Left-class recall decreased from 100% to 66.7%, reflecting a reduction in the systematic Left bias present in the baseline rather than a degradation in discrimination ability. AIDE's errors were distributed across all three classes rather than concentrated in one. These results indicate that the offline advantage of AIDE at SEQ = 5 is preserved in practical operation.

## 4 Discussion

The primary result of this study is that the LLM-optimized classifier achieves 85.03% accuracy with SEQ = 5 repetitions (half the SEQ = 10 required by the baseline system). This translates to almost two-fold reduction in selection time and a corresponding doubling of the ITR at the same repetition count (2.92 vs. 1.43 bits/min at SEQ = 5). The online test corroborated this advantage: AIDE achieved 66.7% accuracy across 18 closed-loop trials compared with 50.0% for the baseline SVM, demonstrating that the offline cross-validation benefit transfers to real-time operation, albeit with a reduction in absolute accuracy relative to the offline estimates.

Auditory ERP-BCIs represent one of the few communication options available to ALS patients in advanced stages of motor paralysis, when gaze-based and motor-imagery systems become inaccessible. The approximately two-fold reduction in selection time demonstrated here translates directly to reduced participant fatigue per session and higher practical communication throughput, both of which are clinically meaningful for end users with severely limited physical endurance. The LLM-assisted optimization paradigm demonstrated here further suggests that BCI pipelines could be rapidly individualized to specific patients by clinical teams without requiring specialized machine learning expertise, lowering the deployment barrier in hospital or home-care settings.

### 4.1 Single-subject design and generalizability

All quantitative results in this study are based on data from a single participant, and direct generalizability to other users cannot be inferred from the present findings. In auditory ERP-BCI research, recruiting and retaining multiple ALS participants across longitudinal recordings is severely constrained by disease progression, participant fatigue, caregiver burden, and the limited pool of patients who are medically stable and sufficiently motivated to complete multi-year EEG protocols. Single-subject designs, however, have a long and well-accepted precedent in BCI research: landmark *n* = 1 case studies include non-invasive EEG-based spelling devices for ALS patients and invasive motor prosthetics (Neuper et al., 2003; Willett et al., 2021). Therefore, the present findings should be interpreted as preliminary proof-of-concept evidence that LLM-based coding assistants can autonomously discover non-obvious individual-specific BCI pipelines, and the primary contribution motivates concrete multi-subject replication.

### 4.2 Offline-to-online transfer

The primary results are from offline cross-validation, which may overestimate online performance due to non-stationarities and real-time processing constraints. Two additional sources of offline–online discrepancy are worth noting. First, the participant's familiarity with the task was relatively limited during the online session compared to the accumulated offline recordings, and reduced habituation to the auditory paradigm may have weakened stimulus-locked cortical responses. Second, a subtle epoch contamination arises specifically at SEQ = 5: because each epoch spans 1000 ms and the next repetition begins before that window closes, the onset-locked response to the sixth stimulus presentation is aliased into the feature vector. This contamination is absent at SEQ = 10, where sufficient silence follows the final repetition before the next trial starts. Inserting a brief post-sequence silent interval could eliminate this artifact and is expected to improve online accuracy at SEQ = 5. This interval can be implemented by replaying one additional stimulus after the fifth repetition solely to fill the 1000 ms window. The 7.3 percentage-point drop in the temporal split condition (77.78%) suggests that the model degrades when applied to data recorded years after training, and periodic recalibration or adaptive learning strategies will be needed for long-term deployment.

### 4.3 Limitations of LLM-based optimization

Two limitations concern the LLM-based optimization paradigm itself. First, the specific optimization trajectory may vary across runs due to the stochastic nature of LLM generation. Although the final model parameters are fully reported (Section 2.7), the optimization process itself is not strictly reproducible. Moreover, because only a single subject was evaluated, it remains unknown whether the same optimization procedure would yield comparable results for other subjects. Second, a constraint violation occurred when a more stringent constraint was imposed on the optimization (e.g., SEQ = 3): the AI violated the constraint to satisfy the accuracy target. Specifically, the AI autonomously increased the repetition count during the search, ultimately reverting to SEQ = 10 to meet the 85% accuracy threshold, rather than finding a solution that respected the specified SEQ = 3 constraint. The AI silently relaxed a hard architectural constraint without reporting infeasibility or seeking human guidance, and the violation was detected only because the human operator actively monitored the optimization output. A plausible cause is that the initial prompt specifying the constraint was effectively “forgotten” over the course of a long optimization process. Two mitigations are possible: external monitoring of the agent's intermediate outputs against the original constraints, and a persistent in-context instruction mechanism such as the one provided by Claude Code. This episode reinforces that human-in-the-loop supervision remains an essential safeguard when LLM-based agents are granted broad optimization authority.

### 4.4 Application for clinical BCI deployment

From a clinical deployment perspective, AIDE could adapt to the individual differences among patients with ALS, and its performance gains, even when modest, could be clinically meaningful. First, the EEG activity patterns of patients with ALS are known to vary both with disease progression and across individuals (Raggi et al., 2010; Riccio et al., 2018; Dukic et al., 2022). Although this is an initial case study, AIDE can be expected to be applied to and optimized for individual patients with ALS, which could be a significant advantage for the clinical deployment of BCI. Second, even the modest improvement in online accuracy from 50.0% to 66.7% observed in this study carries meaningful practical value. More accurate selections mean fewer errors to correct, which increases the amount of effective communication per session. Furthermore, faster and more reliable feedback is relevant to the neuroplasticity-based learning effects that support sustained BCI use (Kleih et al., 2010; Baykara et al., 2016). Even a modest accuracy gain reduces the number of errors the user must correct per session, which compounds across sustained clinical use.

### 4.5 Future directions

Several directions for future work follow from the limitations above. First, multi-subject replication across ALS patients and individuals with other motor disabilities is the most immediate priority, as it would determine whether the LLM-based optimization approach generalizes beyond the single-participant case reported here. Second, extending the Optuna search space to encompass additional feature types, alternative filter architectures, and deep learning classifiers could further improve classification performance and reduce dependency on hand-defined feature modes. Third, developing mechanisms to enforce hard constraints reliably (such as assertion-based scaffolding in the optimization loop or persistent instruction modules within the LLM agent) would directly address the constraint-violation behavior identified in the SEQ = 3 optimization attempt. Fourth, adapting the pipeline to real-time online operation through adaptive recalibration triggered by classification confidence or explicit user feedback would extend the model's utility beyond the current session-level calibration paradigm. Fifth, upon completion of ethics review procedures, depositing the longitudinal EEG dataset in a public neuroimaging repository would enable independent benchmarking and community-level replication of the present findings.

## 5 Conclusion

This study demonstrates that an LLM-based AI coding assistant can autonomously optimize the signal processing pipeline of a three-class auditory ERP-BCI for an ALS patient, achieving reliable classification with half the stimulus repetitions required by the baseline system and without domain-specific algorithmic guidance from the human researcher. Starting from a single natural-language prompt, the AI generated 23 iterative optimization scripts over approximately 24 h and identified an effective pipeline architecture drawing on standard EEG feature constructs selected from a predefined search space, which achieved 85.03% offline cross-validation accuracy at SEQ = 5 (ITR: 2.92 bits/min), compared with 70.11%, 66.90%, and 67.71% for the baseline SVM, LASSO, and LDA at the same repetition count. This yielded a two-fold ITR gain at the same repetition count (2.92 vs. 1.43 bits/min at SEQ = 5). Feature space visualization confirmed that the ZSD + LVD combination chosen by the AI produced visibly separated class clusters, despite no algorithmic guidance from the researcher. Online test confirmed the offline advantage: AIDE achieved 66.7% accuracy vs. 50.0% for the baseline SVM with a markedly more balanced error pattern across the three target classes. While these results are preliminary and based on a single ALS participant, they suggest that LLM-based coding assistants can lower the engineering barrier in BCI pipeline development. Multi-subject replication is the natural next step to assess the generalizability of this optimization methodology.

## Data Availability

The EEG dataset will be made available upon reasonable request to the corresponding author. The analysis code (classifier implementations and figure-generation scripts) is publicly available at https://github.com/mikito-ogino/aide-bci-analysis.

## Supplementary material

Supplementary material S1 Japanese original of the initial optimization prompt.

Supplementary material S2 Complete trajectory of the 23 LLM-generated optimization scripts.

Supplementary material S3 Optuna Bayesian search space and final hyperparameters for v23.

Supplementary material S4 Top channel subsets ranked by mean stratified cross-validation accuracy (channel-subset justification).

Supplementary material S5 Top candidate configurations from the terminal optimization stage (robustness of the final configuration).
