# Supplementary material for: Single-subject auditory ERP-BCI performance enhancement in ALS via an AI coding assistant prompt
Source: Front Hum Neurosci. 2026 Jul 1;20:1869918. doi: 10.3389/fnhum.2026.1869918 (PMC13369013; doi:10.3389/fnhum.2026.1869918)
Supplement: Supplementary material S1 — Japanese original of the initial optimization prompt. [file Data_Sheet_1.pdf]

Supplementary Material

S1. Japanese Original of the Initial Optimization Prompt

The following is the verbatim Japanese prompt entered by the researcher on 2026-04-10 at approximately 10:51 JST to initiate the AI-driven optimization described in the main text. The prompt refers to the existing BCI classification script and its printed accuracy metric as the optimization target.

Listing 1: Initial prompt entered by the researcher (Japanese original).

```
[既存のBCI分類スクリプト]をコピーして、そのスクリプト内
    でprint表示しているMean accuracy（k分割交差検証の平
    均正解率）を最大化するようにauditory erp bciの解析
    を行なって。データはdataの中の全データが対象。既存
    スクリプトでどのように解析を行なっているのかをまず
    は調べて、SEQ=5（1試行あたりの刺激反復回数）は固定
    で解析して。SEQ=10だと高精度になるが、遅いので、ス
    ピードを早くするのが目的。Mean accuracyの出し方も固
    定し、mean accuracyが0.85を越えるまで解析をし続け
    て。pip installは自動で行なって。途中止まらないよう
    に、全ての実行を許可して。精度の向上経過はタイムス
    タンプと使った手法と共にcsvに出力して。
```

An English translation of this prompt is provided in the main text.

S2. Full Optimization Trajectory (23 Scripts)

The main text reports six representative milestones in the AI-driven optimization (Table 1). Table 1 below provides the complete trajectory across all 23 successive optimization scripts generated by Claude Code. For each script, we report the version number, the number of candidate configurations evaluated, the best mean stratified cross-validation accuracy reached within that script, the timestamp at which that script began executing, and a short label of the dominant algorithmic change introduced relative to the preceding version.

Table 1: Complete trajectory of the 23 LLM-generated optimization scripts. Best accuracy is the maximum mean stratified cross-validation accuracy ($k \in \{3, 4\}$, 10 random seeds, undersampled training folds) attained by any candidate configuration evaluated within that script.

v#	Start (JST)	Trials	Best acc.	Key change
2	04-10 10:56	306	74.05%	Baseline SVC + Optuna scaffolding
3	04-10 11:13	1334	75.68%	Channel subset & downsampling search
4	04-10 11:23	428	78.03%	LDA classifier introduced
5	04-10 11:36	652	77.80%	Scaled SVC, pairwise difference features
6	04-10 12:03	921	79.75%	LDA + lsqr + auto shrinkage
7	04-10 14:08	1150	79.82%	LDA + eigen + auto shrinkage
8	04-10 14:26	5	—	XGBoost (failed; aborted run)
9	04-10 14:40	1241	80.08%	LDA eigen on z-scored differences
10	04-10 16:02	678	79.59%	Window-size and downsample tuning
11	04-10 16:18	855	81.38%	Mean+variance difference (MVD)
12	04-10 16:39	2896	81.30%	Artifact rejection at 100 μV
13	04-10 16:43	1840	80.65%	Tri-band + scaled logistic regression
14	04-10 16:47	2706	80.67%	Combined feature search
15	04-10 19:01	4982	80.67%	Two-band sweep (low/high cutoff grid)
16	04-10 19:03	5523	82.82%	Wide MVD search w/ shrinkage tuning
17	04-10 19:07	4137	83.21%	Two-band approach + gamma VD
18	04-10 22:58	2799	84.06%	Channel subset F3,Fz,F4,C3,C4 fixed
19	04-11 07:43	1709	83.25%	Independent low/high mode search
20	04-11 10:16	1125	83.25%	Restricted shrinkage & cutoff bounds
21	04-11 10:31	41	83.11%	Targeted parameter check
22	04-11 10:48	537	84.59%	Optuna refinement, eigen solver
23	04-11 10:53	580	**85.03%**	Sub-delta ZSD + gamma LVD + lsqr

Across the 23 scripts (excluding the failed v8 XGBoost run), the AI evaluated 36,448 distinct candidate configurations. Accuracy increased from 74.05% (v2) to 85.03% (v23) over approximately 30 wall-clock hours, of which roughly 24 hours involved active optimization (the remaining time was spent waiting for the daily API token reset).

S3. Bayesian Search Space and Final Hyperparameters

The terminal optimization script (v23) used Optuna with Tree-structured Parzen Estimator (TPE) sampling to search a multi-dimensional configuration space. Table 2 lists each searched dimension, its prior range, and the value selected at the optimum. The categorical dimensions m_1, m_2 correspond to feature-extraction modes computed on the per-pair averaged ERPs: *zsd* (z-scored mean difference), *vd* (variance difference), *mvd* (mean+variance difference, concatenated), *zvd* (z-scored variance difference), *log_vd* (log-scale variance difference), and *mvd_log* (mean+log-variance difference, concatenated). The channel-set dictionary contained 24 candidate subsets of the 8-channel montage; the optimum (`01235`) corresponds to {F3, Fz, F4, C3, C4}.

S4. Channel-Subset Justification

The five-channel subset {F3, Fz, F4, C3, C4} (index code `01235`) was identified by the AI as superior to all other subsets evaluated during the 23-script optimization. We retrospectively aggregated all 31,401 candidate configurations from the 23 scripts that explicitly tagged a channel subset, and computed the best mean stratified cross-validation accuracy attained by each subset across all parameter combinations. Table 3 ranks the top 10 subsets by their best accuracy. The selected `01235` subset attained the global maximum (85.03%) and was evaluated 2,027 times, providing strong evidence that the choice is not an artifact of limited exploration. Notably, full 8-

Table 2: Optuna search space for v23 and the parameter values selected at the global optimum (mean stratified k-fold accuracy 85.03%).

Parameter	Search range / candidates	Selected
Preprocessing		
use_car	{True, False}	False
avg_mode	{mean, trimmed, weighted}	mean
ch (channel subset)	24 named subsets of 8 channels	01235 (F3,Fz,F4,C3,C4)
ds (downsample)	{2, 5, 10, 25, 50}	10
bl (baseline samples)	integer $\in [0, 3]$	0
Two-band filtering		
lc1 (low cutoff, band 1)	uniform $[0.08, 0.35]$ Hz	0.211 Hz
hc1 (high cutoff, band 1)	uniform $[2.0, 15.0]$ Hz	2.007 Hz
lc2 (low cutoff, band 2)	uniform $[0.08, 0.35]$ Hz	0.208 Hz
hc2 (high cutoff, band 2)	uniform $[20.0, 120.0]$ Hz	39.045 Hz
Feature extraction		
m1 (mode, low band)	{zsd, vd, mvd, zvd, log_vd, mvd_log}	zsd
m2 (mode, high band)	{zsd, vd, mvd, zvd, log_vd, mvd_log}	log_vd
Classifier (LDA)		
solver	{eigen, lsqr}	lsqr
shrinkage	uniform $[0.35, 0.95]$	0.726

channel montages (`all8`, `0123456`) ranked below `01235`, indicating that the LDA classifier benefits from removing parietal (P3, P4) and central midline (Cz) channels, which contributed redundant or noise-dominated dimensions relative to the small training-set size ($n = 189$).

S5. Robustness of the Final Configuration

A natural concern with stochastic Bayesian optimization is whether the reported optimum (85.03%) is a fortuitous outlier produced by a single lucky parameter combination, or a stable region of the search space. To address

Table 3: Top 10 channel subsets ranked by best mean stratified cross-validation accuracy across all candidate configurations evaluated in the 23-script optimization. "n trials" counts the number of times each subset was sampled. The selected subset (`01235`) is highlighted.

Subset (index)	Channels	n trials	Best acc.	Median acc.
**01235**	F3, Fz, F4, C3, C4	2027	**85.03%**	82.84%
0125	F3, Fz, F4, C4	6485	84.40%	79.93%
0135	F3, Fz, C3, C4	37	83.31%	82.65%
01356	F3, Fz, C3, C4, P3	1	82.66%	82.66%
012345	F3, Fz, F4, C3, Cz, C4	361	82.63%	78.31%
012356	F3, Fz, F4, C3, C4, P3	36	82.63%	81.89%
0123	F3, Fz, F4, C3	4142	82.59%	77.24%
all8	all 8 channels	2478	82.31%	77.89%
0125678	F3, Fz, F4, C4, P3, P4	35	81.67%	80.24%
015	F3, Fz, C4	1	81.87%	81.87%

this, we examined the distribution of accuracy across all 580 candidate configurations evaluated in the terminal script (v23). The top 5% of v23 trials (29 configurations) all exceeded 84.5% mean accuracy, and the top 10 configurations are listed in Table 4. All ten share the same categorical structure: the `01235` channel subset, `lsqr` solver, downsampling factor 10, no CAR, zero-sample baseline, mean averaging across repetitions, and the two-mode feature pair (`zsd`, `log_vd`). The continuous parameters cluster tightly: shrinkage $\in [0.685, 0.774]$, low-band cutoff $\in [0.20, 0.22]$ Hz, low-band high cutoff $\in [2.00, 2.76]$ Hz, high-band cutoff $\in [38.6, 40.7]$ Hz. The convergence of these top configurations to a single architecture indicates that the AI identified a robust minimum rather than a narrow accidental peak; small perturbations of the continuous hyperparameters within the cluster preserve the $84-85\%$ accuracy plateau.

All 10 configurations use channel subset `01235` (F3, Fz, F4, C3, C4), solver `lsqr`, downsampling factor 10, no CAR, zero-sample baseline, mean averaging across stimulus repetitions, low-band feature mode `zsd`, and high-band feature mode `log_vd`. The remaining low-band cutoff lc_1 ranged within $[0.205, 0.218]$ Hz and the high-band cutoff lc_2 within $[0.176, 0.211]$ Hz, both well inside the prior ranges specified in Table 2.

Table 4: Top 10 candidate configurations from the terminal optimization script (v23). All share the same categorical architecture; only the continuous parameters (shrinkage sh, filter cutoffs hc_1, hc_2) vary within narrow ranges.

Rank	Mean acc.	Shrinkage	Low-band hc_1 (Hz)	High-band hc_2 (Hz)
1	**85.03%**	0.726	2.007	39.045
2	84.99%	0.720	2.002	39.209
3	84.81%	0.713	2.440	38.934
4	84.81%	0.774	2.349	39.913
5	84.79%	0.740	2.018	38.590
6	84.75%	0.720	2.238	40.213
7	84.70%	0.707	2.759	39.245
8	84.70%	0.685	2.252	40.410
9	84.70%	0.704	2.274	38.633
10	84.70%	0.713	2.404	40.700
